# Non-Invasive Indirect Markers of Liver Fibrosis after Interferon-Free Treatment for Hepatitis C

**DOI:** 10.3390/jcm10173951

**Published:** 2021-08-31

**Authors:** Dagmara Przekop, Jakub Klapaczynski, Agnieszka Grytczuk, Ewa Gruszewska, Andrzej Gietka, Anatol Panasiuk, Slawomir Golaszewski, Bogdan Cylwik, Lech Chrostek

**Affiliations:** 1 Diagnostics-Experimental Center of Sexually Transmissible Diseases, 15-879 Bialystok, Poland; dagmara.przekop@o2.pl; 2 Department of Internal Diseases and Hepatology, Central Clinical Hospital of Ministry of Home Affairs and Administration, 02-507 Warszawa, Poland; jklapaczynski@hepatolodzy.pl (J.K.); andrzejgietka@o2.pl (A.G.); 3 Department of Laboratory Diagnostics, University Clinical Hospital in Bialystok, 15-540 Bialystok, Poland; agnieszka.grytczuk@onet.eu; 4 Department of Biochemical Diagnostics, Medical University of Bialystok, 15-269 Bialystok, Poland; ewa.gruszewska@umb.edu.pl; 5 Department of Internal Diseases and Gastroenterology, Provincial Welded Hospital in Bialystok, 15-950 Bialystok, Poland; anatol@panasiuk.pl; 6 Department of Clinical Medicine, Medical University of Bialystok, 15-254 Bialystok, Poland; 7 Department of General, Minimally Invasive and Oncological Surgery, Provincial Welded Hospital in Bialystok, 15-950 Bialystok, Poland; slawekg1966@gmail.com; 8 Department of Pediatric Laboratory Diagnostics, Medical University of Bialystok, 15-274 Bialystok, Poland; cylwikb@umb.edu.pl

**Keywords:** hepatitis C, 3D regimen, HCV eradication, non-invasive fibrosis markers

## Abstract

The effectiveness of interferon-free therapy during the course of HCV infection has already been confirmed. Liver fibrosis can be assessed in several ways, from biopsies to imaging tests. The present study evaluates the usefulness of non-invasive indirect biomarkers of liver fibrosis (APRI, GAPRI, FORNS, FIB-4, the AP index and HUI score) as markers of the effective treatment of HCV with the 3D regimen. Blood samples were collected from 70 patients suffering from chronic hepatitis C. Patients received the 3D AbbVie regimen for hepatitis C. All patients had HCV genotype 1b. The APRI, GAPRI, FIB-4, FORNS, HUI and AP index (age–platelet score) values were calculated with their respective algorithms. The stage of fibrosis was evaluated on the basis of a liver biopsy and confirmed by FibroScan-based transient elastography. An undetectable level of HCV RNA after 12 weeks of treatment with the 3D regimen indicates 100% eradication of hepatitis C virus. After the treatment, non-invasive indirect markers of liver fibrosis achieved levels below the limit for significant fibrosis, Thus, non-invasive indirect biomarkers of hepatic fibrosis failed to detect the presence of significant fibrosis, which was proved in histopathological examination. However, the eradication of hepatitis C virus by means of the 3D regimen treatment does not mean that patients were completely cured.

## 1. Introduction

The treatment of chronic hepatitis C virus (HCV) infection has been revolutionized in recent years [1]. The introduction of direct-acting antiviral agents (DAAs) that target different viral functions, instead of interferon-based regimens, has increased the rates of sustained virologic response after 12 weeks of therapy (SVR12) in HCV-infected patients without cirrhosis or with compensated cirrhosis to 94–97% [2]. The new therapeutic regimen for the treatment of chronic HCV infection consists of two pills: one tablet called Viekirax/Exviera in Europe, comprising of the NS5A inhibitor ombitasvir, the protease inhibitor paritaprevir and cytochrome P3A inhibitor ritonavir; and a second tablet with non-nucleoside polymerase inhibitor dasabuvir (DSV) [3]. The combination of this three-drug regimen plus dasabuvir was developed by AbbVie and Enanta (AbbVie, Inc., North Chicago, IL, USA, and Enanta Pharmaceuticals, Inc., Watertown, MA, USA) and, together, they are known as the 3D regimen [4]. This is the new standard of care for patients infected with HCV and is currently approved for the treatment of HCV genotype 1 (with DSV) and genotype 4 (without DSV) in the USA and Europe [5]. A regulatory committee has recommended that the European Medicines Agency (EMA) approve AbbVie’s 3D regimen, with or without ribavirin, to treat genotypes 1 and 4 of the hepatitis C virus. The most recent recommendations on the treatment of hepatitis C were prepared in 2018 [6].

The traditional and benchmark tool to evaluate liver fibrosis and cirrhosis is liver biopsy. However, this method has significant limitations—among many others, its invasiveness, risk of complications and inability to monitor disease progression [7]. For this reason, several noninvasive serum markers have been developed on the basis of serum tests. Their superiority to liver biopsy rests on their non-invasiveness and the possibility of repeated use, as liver fibrosis is a dynamic process. Changing levels can be monitored with laboratory tests, which can be pooled in panels known as non-invasive serum biomarkers of liver fibrosis [8].

In this study, we evaluated six indirect, non-invasive biomarkers of liver fibrosis (APRI, GAPRI, Forn’s, FIB-4, Age-Platelet and HUI score) as markers of the effective treatment of HCV with the 3D regimen. Additionally, we assessed the difference in fibrosis reversal between patients who only received the 3D regimen (the naïve group) and those who had been pre-treated prior to the administration of interferon-free therapy (the experienced group).

## 2. Materials and Methods

### 2.1. Patients

The study included 70 patients suffering from the hepatitis C virus: 32 women with a mean age of 54 (range: 27–74 y) and 38 men with a mean age of 48 (range: 24–81 y). The study recruited patients referred by GPs with diagnosed HCV infection. A total of 39 patients did not receive any treatment before the study and were called the “naïve” group, and 31 patients received treatment before the 3D AbbVie regimen and were named the “experienced” group. Pre-treatment therapy included pegylated interferon-α and ribavirin (PEG INF + RBV). Patients were interviewed about drinking alcohol and none of them abused. Their declared weekly alcohol consumption was less than 2 standard drinks (1 standard drink was defined as 14 g of pure alcohol). All subjects gave informed consent before they participated in the study. This study was in accordance with the Helsinki Declaration and was approved by the Bioethical Committee at the Medical University of Bialystok.

### 2.2. Blood Sampling

Blood samples from each patient were collected from a peripheral vein. After centrifugation, sera were collected in 2 tubes and stored at −86 °C until assayed. Besides serum, a part of each blood sample was collected in tubes containing 3.8% liquid sodium citrate and EDTA-2.

### 2.3. Diagnosis

The diagnosis was based on clinical data: signs, symptoms, physical exams and abdominal ultrasound or abdominal CT, laboratory tests (the biochemical liver panel: PLT, MCV, AST, ALT, GGT, albumin, bilirubin and cholesterol) and a liver biopsy. Liver biopsies were performed percutaneously using ultrasound guidance. Liver biopsies were performed using Hepafix 1.6 mm (B Braun Melsungen AG, Melsungen, Germany) with two passages. The obtained material was 12–26 mm long and contained 6–14 portal spaces. Liver biopsies were assessed by a histopathologist specializing in hepatology. Each liver biopsy was accompanied by a protocol with complete clinical information about the patient. Histopathological examination showed no other significant changes apart from fibrosis and inflammation of the liver tissue.

The obtained 20 mm biopsy specimens included 1–12 portal tracts. To evaluate the stage of fibrosis, we used METAVIR classification on the basis of the results of elastography. The FibroScan scoring system enabled us to present fibrosis results as not only total but also transitional scores (F0–F1, F1–F2, F2–F3 and F3–F4). We assumed that such subpopulations of patients would enable fibrosis to be more accurately assessed. Significant fibrosis and cirrhosis were defined as stages F2–F4 and F4, respectively. Each patient also had vibration-controlled transient elastography (VCTE) utilizing FibroScan (FibroScan 502 Touch; EchoSens, Paris, France) performed by physicians trained and certified by the manufacturer. The biopsies were evaluated by the same histopathologist. The liver stiffness measurement (LSM) was expressed in kilopascals (kPa). Before examination, each patient was asked not to consume any liquids or solids for a minimum of 3 h before the test. The scan typically took 10 to 15 min. To confirm the diagnosis of hepatitis C, anti-HCV tests were performed by a third-generation EIA (Ortho-Diagnostic Systems).

### 2.4. Treatment

A total of 70 HCV-infected patients received the 3D AbbVie regimen for hepatitis C. The 3D regimen (marketed as Viekirax/Exviera in Europe) consists of the HCV protease inhibitor paritaprevir (75 mg) boosted with ritonavir (50 mg), the NS5A inhibitor ombitasvir (12.5 mg) and the NS5B polymerase inhibitor dasabuvir (250 mg). Patients received a 12-week regimen consisting of a single tablet co-formulation of ritonavir-boosted paritaprevir and ombitasvir (at a once daily dose of 150 mg paritaprevir, 100 mg ritonavir and 25 mg ombitasvir) and dasabuvir (250 mg twice daily). The primary goal was a sustained virological response 12 weeks after the end of treatment (SVR12). All examinations (including the biopsy, LSM (liver stiffness measure), HCV RNA detection and quantification and laboratory tests for biomarkers) were conducted twice: before the 3D regimen (point 0) and after 12 weeks (point 12).

### 2.5. Laboratory Testing

AST, ALT, GGT, albumin, cholesterol and bilirubin were determined on a Cobas c501 Analyzer (Hitachi, Tokyo, Japan). The PLT count was measured on a Sysmex XS-800i (Sysmex Corporation, Singapore).

Calculations of non-invasive indirect fibrosis biomarkers:

APRI = (AST [IU/L]/ULN)/(PLT [10^9^/L]) * 100 [9]
*ULN, AST upper level of normal (50 IU/L)
GAPRI = (GGT [IU/L]/ULN/(PLT [10^9^/L]) * 100 [10]
*ULN, GGT upper level of normal (40 IU/L for women, 75 IU/L for men)
FIB-4 = (age [y] * AST [IU/L])/(PLT [10^9^/L] * √(ALT [IU/L]) [11]
Forn’s index = 7.811 − 3.131 ln (PLT [10^9^/L]) + 0.781 ln (GGT [IU/L]) + 3.467 ln (age) − 0.014 (cholesterol [mg/dL]) [12]
AP index = age [y] + PLT (age: <30 = 0; 30–39 = 1; 40–49 = 2; 50–59 = 3; 60–69 = 4; ≥70 = 50, PLT [× 10^9^/L]: ≥225 = 0; 200–224 = 1; 175–199 = 2; 150–174 = 3; 125–149 = 4; <125 = 5) [13]
HUI score = 3.138 + 0.167 × BMI + 0.088 × bilirubin [mg/dL] − 0.151 × albumin [g/dL] − 0.019 × PLT [10^9^/L] [14].

### 2.6. Detection and Quantification of HCV RNA

The detection of HCV (viremia) and quantification of HCV genotypes was conducted with a qualitative reverse transcriptase-polymerase chain reaction (RT-PCR) using a fully automated Cobas AmpliPrep/Cobas TaqMan HCV, test version 2.0 (CAP/CTM HCV; Roche Molecular Systems, Pleasanton, CA). In this system, the Cobas AmpliPrep performs an automated extraction and the Cobas TaqMan device performs automated real-time PCR amplification and quantification. The lower limit of detection of HCV is 15 IU/mL. All patients had HCV genotype 1b.

### 2.7. Statistical Analysis

The normality of distribution was ascertained by means of the Kolmogorov–Smirnov test with the Lilliefors correction. The analysis revealed that the distribution of tests did not follow a normal distribution (*p* < 0.05). To compare two related samples (point 0 and point 12) we used the Wilcoxon signed-rank test. The differences between the experienced and naïve groups were evaluated using the Mann–Whitney U test. The correlation between FibroScan results and non-invasive indirect markers of liver fibrosis variables was assessed using Spearman’s rank correlation coefficient. We considered *p*-values of <0.05 as statistically significant.

## 3. Results

There was no significant difference (*p* = 0.917) between viremia in experienced and naïve patients at the start of treatment (point 0) and after 12 weeks of treatment (point 12) (Table 1). There was also no significant difference in liver stiffness measurement between naïve and experienced patients at point 0 of the study (*p* = 0.376) and at point 12 (*p* = 0.390). Interestingly, there were significant differences in liver stiffness in the group of experienced patients between point 0 and 12 of the study (*p* = 0.043) and no significant difference in the naïve group between point 0 and 12 (*p* = 0.081).

The activities of liver enzymes (AST, ALT and GGT) significantly decreased after 12 weeks of treatment with AbbVie’s 3D regimen in the naïve and experienced groups (*p* < 0.001 for all comparisons) (Table 2). The concentrations of total bilirubin and cholesterol did not change after 12 weeks of AbbVie’s 3D regimen in the naïve and experienced groups of HCV patients (*p* > 0.05 for all comparisons). The number of platelets significantly increased in the naïve patients after 12 weeks of AbbVie’s 3D regimen (*p* = 0.002). We noticed a significantly increased concentration of albumin in the naïve patients after AbbVie’s 3D regimen (*p* = 0.038).

After 12 weeks of AbbVie’s 3D regimen, we observed an increase in the number of low scores on the METAVIR scale, as follows: the prevalence of the score F0–F1 increased from 47.1 to 60% after treatment, the score of F1 increased from 1.4 to 7.1% and the score of F1–F2 from 11.4 to 14.3%. There was a parallel increase in the prevalence of the highest stage of liver fibrosis: from 21.4% before treatment to 11.4% after AbbVie’s 3D regimen (Table 3).

**Figure 1:**
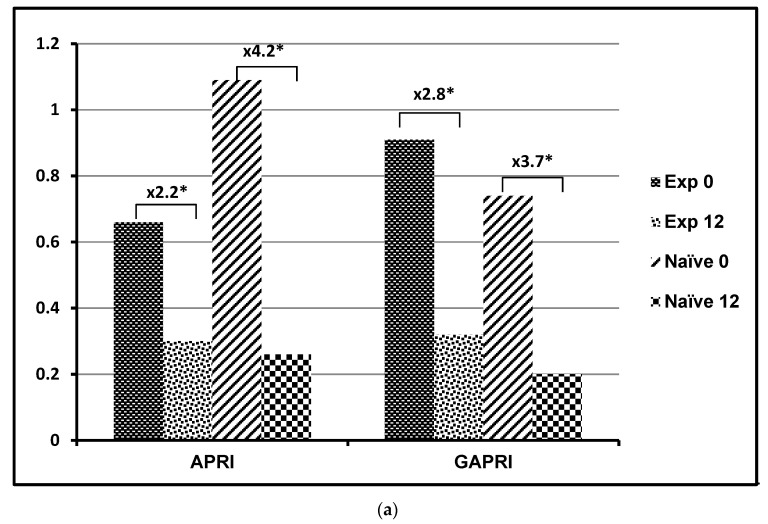

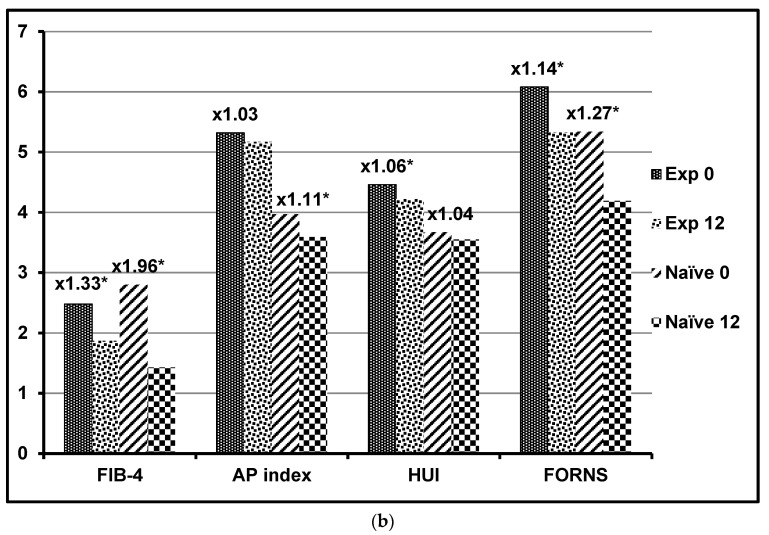
The value of non-invasive indirect biomarkers of liver fibrosis before and after 3D regimen treatment of HCV in the experienced and naïve groups of patients. (**a**) tests with multiple value changes (**b**) tests with little or no change in value. Exp—treatment experienced group; Naïve—treatment naïve group; 0—start of treatment; 12—12 weeks after start of treatment. The numbers show multiple increases in the groups from baseline (point 0) after 12 weeks of treatment; * statistically significant differences between point 12 and 0 in experienced and naïve group.

The value of APRI decreased significantly, by about 2.2 times, after AbbVie’s 3D regimen in the naïve patients (Z = 4.379; *p* < 0.001) and by about 4.2 times in the experienced group (Z = 5.227; *p* < 0.001). The value of GAPRI similarly decreased by about 2.8 times after AbbVie’s 3D regimen in the naïve patients (Z = 4.508; *p* < 0.001) and by about 3.7 times in the experienced group (Z = 5.303; *p* < 0.001) (Figure 1a). FIB-4 and Forn’s index also significantly decreased in the naïve and experienced patients after AbbVie’s 3D regimen, but the differences were not high as for APRI and GAPRI (Figure 1b). In the experienced group, FIB-4 decreased by around 33% (Z = 2.887; *p* = 0.004) and Forn’s index by 14% (Z = 3.708; *p* < 0.001). In the naïve group, FIB-4 decreased by around 96% (Z = 4.413; *p* < 0.001) and Forn’s index by around 14% (Z = 5.227; *p* < 0.001). The AP index decreased only in naïve patients, by around 11% (Z = 2.578; *p* = 0.009), and HUI only in the experienced group, by around 6% (Z = 1.978; *p* = 0.048).

When compared, the values of tested biomarkers between the naïve and experienced groups at point 0 and point 12 of the treatment only differed on the AP index at point 0, but by the end of treatment there were significant differences for FIB-4, the AP index and FORNS (Table 4).

When variations of non-invasive biomarkers from week 0 to week 12 were compared, significant trends were observed for APRI and GAPRI. This implies that values of these tests below the cut-off for significant fibrosis are more likely after treatment in both groups.

All non-invasive indirect biomarkers of liver fibrosis correlated moderate with FibroScan results at the beginning of the treatment (*p* < 0.001 for all indicators) but weakly at the end of the therapy (R < 0.4), except for GAPRI, which correlated strongly at the end of therapy (Table 5).

**Figure 2:**
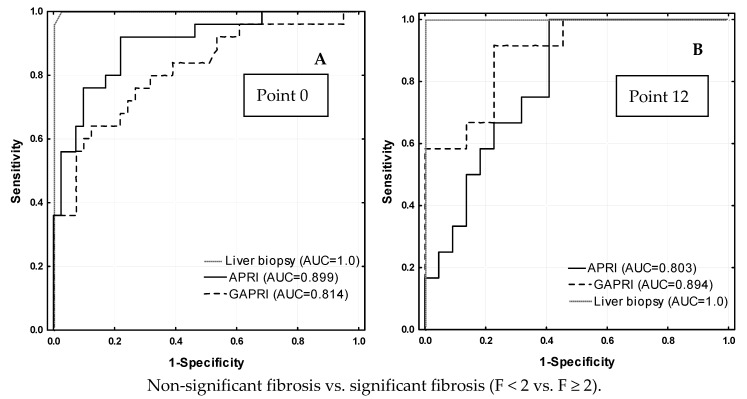

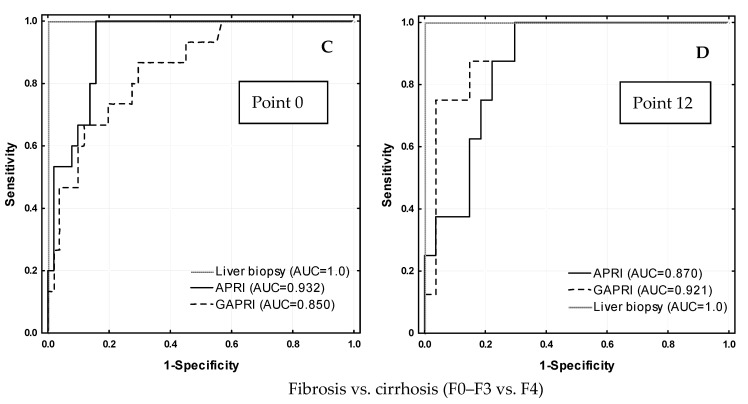
The comparison ROC curves for BIOPSY, APRI and GAPRI between non-significant fibrosis (F < 2) and significant fibrosis (F ≥ 2) at point 0 (**A**) and 12 (**B**), and between fibrosis (F0-F3) and cirrhosis (F = 4) at point 0 (**C**) and 12 (**D**).

The diagnostic power of non-invasive fibrosis tests which values change are presented in Figure 2. As expected, the AUCs at point 0 for APRI and GAPRI for the differentiation of significant and non-significant fibrosis were significantly lower than those for the FibroScan and biopsy (*p* = 0.019 and *p* < 0.001, respectively) (Figure 2A). At time 12, the AUCs for these tests did not differ significantly (Figure 2B). However, the AUCs for APRI and GAPRI for the differentiation of fibrosis and cirrhosis were significantly lower than those for the FibroScan and biopsy at point 0 (*p* = 0.021 for APRI and *p* < 0.001 for GAPRI) (Figure 2C) and 12 (*p* < 0.001 for APRI and GAPRI) (Figure 2D).

## 4. Discussion

This study verified that a 3D regimen efficiently eliminates HCV RNA in all tested patients. It confirms the effectiveness of 3D therapy and that testing viremia by means of HCV RNA is the best way to prove therapeutic success. It implies that sustained virologic response rates at 12 weeks post-treatment (SVR12) were achieved in pretreatment and non-pretreatment patients, and non-cirrhotic and cirrhotic patients with genotype 1b HCV [1,2,3,20,21,22,23]. Undetectable levels of HCV RNA after 12 weeks of treatment prove 100% eradication of the hepatitis C virus. However, this does not mean that patients were completely cured. In order to verify this hypothesis, we performed another liver biopsy. This was performed after treatment, at the same time as the elastography and non-invasive indicators of liver fibrosis were assessed. Looking at the results of the biopsy after 12 weeks of treatment, it is possible to draw conclusions about the need for further liver fibrosis therapy. The proportion of cases with significant fibrosis (≥F2) only decreased from 30% at the beginning of therapy to 18.5% at the end of medication. Therefore, after efficient eradication of HCV infection with the 3D regimen, there is still a need to continue medication to reverse fibrosis—a change from causal to symptomatic treatment. It also implies that 12 weeks after treatment with the 3D regimen is too short a time for full regression of fibrosis, especially in cases with cirrhosis (F4), in which case a reduction in cirrhosis was observed in half of the patients (decrease from 21.4 to 11.4%). The other patients with cirrhosis experienced no change in the severity of the disease. Our results indicate that only two tests, APRI and GAPRI, reached values lower than the cut-off for significant fibrosis in the experienced and naïve groups. These results are inconsistent with the results of the elastography and biopsy. Therefore, these tests cannot be used as predictors for fibrosis change. An indirect proof of this is the variation of these tests from week 0 to week 12, in which a chi-squared test for trend analysis showed a significant decreasing trend in APRI and GAPRI values over time in both study groups The analysis of the ROC curves shows that both APRI and GAPRI can be useful in the diagnosis of liver fibrosis, as AUCs obtained values above 0.8. However, the diagnostic power of both of these tests was significantly lower than that of the liver biopsy and FibroScan for discriminating between non-significant and significant fibrosis. While at point 0 APRI showed higher diagnostic accuracy, at point 12 GAPRI had a higher accuracy, both for discriminating non-significant from significant fibrosis and for cirrhosis fibrosis.

Two systems were used to assess fibrosis: the first, the METAVIR scoring system, which evaluates fibrosis by histopathological examination (biopsy) [15,16]; the second, vibration-controlled transient elastography (VCTE) utilizing FibroScan, which measures liver stiffness (LSM) in a volume a hundredfold greater than that obtained by needle biopsy [17,18]. Both techniques evaluate morphological changes in liver parenchyma. Parallel to the morphological studies of liver parenchyma, we also examined changes in non-invasive indirect biomarkers of liver fibrosis. The formulae of these tests of hepatic fibrosis involve enzymes (both aminotransferases and γ-glutamyl transferase) that are not only indicators of hepatocyte damage but also of the inflammation of liver tissue [19]. These are APRI (containing AST), GAPRI (containing GGT) and FIB-4 (containing AST and ALT). In our opinion, the presence of these enzymes in the biomarker formulae are the main cause of the highest changes in APRI, GAPRI and FIB-4 values after 12 weeks of treatment. This implies that after that time the inflammatory process in the liver was extinguished. At the start of treatment, when inflammation was the strongest, all the biomarkers strongly correlated with liver stiffness (FibroScan) and after 12 weeks of treatment this correlation decreased with the exception of the correlation with GAPRI, which increased. It is very satisfying that the value of tested non-invasive indirect markers of liver fibrosis after 12 weeks of treatment reached a value below the cut off for significant fibrosis, especially in the naïve group [9,10,11,12,24,25]. The mean values of APRI and GAPRI at the end of treatment in both the naïve and experienced groups were below the cut-off values for significant fibrosis [9,10]. FIB-4, FORNS and the AP index obtained these values only in the naïve group [11,24,25]. It is noteworthy that the mean values of LSM (FibroScan) in each subgroup did not cross the border for insignificant fibrosis, which is 7.1 kPa [26]. These values track the range of severe fibrosis (more than 9.6 kPa and below 12.5kPa). This implies a greater harmony with liver biopsy than with the other non-invasive biomarkers.

We also noticed that the biomarker values after 12 weeks of treatment were generally lower in the naïve than the experienced group, with statistically significant differences for FIB-4, FORNS and the AP index. However, this was not dependent on the mean values of markers at the start of the treatment, as for APRI and FIB-4 the mean values at the start of therapy were higher in the naïve group than the experienced group, but for the remaining indicators there was an inverse relationship. Generally, the non-invasive indirect biomarkers of liver fibrosis that contain other enzyme components, i.e., cholesterol (FORNS), bilirubin and albumin (HUI), or platelets alone (AP index), changed only marginally. We can see that the concentration of total cholesterol did not change after medication. This is one of the components of the FORNS formula, and therefore the changes in this marker are related to changes in GGT and platelets. However, platelets changed only for the naïve group. There were also no changes in bilirubin concentration, which is a component of the HUI formula. The changes in HUI value could have been related to albumin and platelets. However, albumin changed only in the experienced group and thus that change could explain the change in HUI in this group of patients only. The change in the AP index in the naïve group only was linked with the change in the number of platelets, which occurred in this group of patients only. To summarize, the changes in the values of the biomarkers of liver fibrosis can be explained by changes at the level of their components.

The time span of observation is a limitation of this study. Looking at these results, we are aware that to observe a fibrosis regression a follow-up period longer than 12 weeks would be needed. Beyond this, it is expected that DAAs would allow HCV eradication, while liver fibrosis regression takes longer, despite the clear normalization of liver tests on the part of non-invasive fibrosis markers.

## 5. Conclusions

In conclusion, we can state that after the treatment of hepatitis C virus with the 3D regimen, the values of non-invasive indirect markers of liver fibrosis achieved levels below the limit for significant fibrosis. Though the non-invasive indirect biomarkers of hepatic fibrosis were highly correlated with stiffness, they failed to indicate the presence of significant fibrosis, which was proved in histopathological examination by means of a liver biopsy and FibroScan. This implies that these markers did not provide reliable data.

## Figures and Tables

**Table 1:** Characteristics of HCV-infected patients.

	Sex/ Week of Treatment	Treatment	Median	Minimum	Maximum	*p*
Sex	Women *n* = 32	Experienced	62	35	69	0.056
		Naïve	46	27	74	
	Men *n* = 38	Experienced	50	30	81	0.399
		Naïve	43	24	77	
BMI [kg/m^2^]	Women *n* = 32	Experienced	27.0	22.5	43.1	0.027 *
		Naïve	23.6	20.4	30.8	
	Men *n* = 38	Experienced	26.2	12.9	30.5	0.690
		Naïve	26.0	22,3	33.6	
Viremia [IU/mL]	0	Experienced	853,000	20,300	8,507,396	0.917 ^a^
	12		ND	ND	ND	
	0	Naïve	943,000	31	111,400,000	
	12		ND	ND	ND	
FibroScan [kPa]	0	Experienced	8.4	3.7	32.8	0.273 ^a^ 0.043 ^b,^*
	12		7.0	3.3	44.3	
	0	Naïve	6.6	2.8	69.1	0.081 ^b^ 0.390 ^c^
	12		6.6	3.5	36.3	

Notes: “^a^” in superscript—the comparison between the experienced and naïve groups at point 0; “^b^” in superscript—the comparison between point 0 and 12 in the experienced and the naïve group; “^c^” in superscript—the comparison between the experienced and the naïve group at point 12. * statistically significant difference in Mann–Whitney U test. Abbreviations: W, women; M, men; 0, start of the 3D regimen treatment; 12, after 12 weeks of the 3D regimen treatment; BMI, body mass index; ND, not detectable viremia.

**Table 2:** Changes in biochemical parameters in HCV treatment patients.

	Treatment	Week	Median	Minimum	Maximum	*p*
ALT [IU/L]	Experienced *n* = 31	0	60	14	94	0.000 *
		12	19	5	39	
	Naïve *n* = 39	0	56	17	290	0.000 *
		12	20	10	44	
AST [IU/L]	Experienced *n* = 31	0	47	26	96	0.000 *
		12	21	14	58	
	Naïve *n* = 39	0	45	19	309	0.000 *
		12	21	11	41	
GGT [IU/L]	Experienced *n* = 31	0	64	14	365	0.000 *
		12	25	10	70	
	Naïve *n* = 39	0	47	11	215	0.000 *
		12	19	9	32	
BIL [µmol/L]	Experienced *n* = 31	0	10.3	4.3	35.9	0.304
		12	10.3	4.4	17.1	
	Naïve *n* = 39	0	8.6	3.8	31.5	0.667
		12	7.7	4.4	41.7	
PLT [×10^9^]	Experienced *n* = 31	0	189	64	450	0.299
		12	187	44	427	
	Naïve *n* = 39	0	216	37	431	0.002 *
		12	229	64	409	
ALB [g/L]	Experienced *n* = 31	0	39	26.7	5	0.038 *
		12	43.0	36.0	47.8	
	Naïve *n* = 39	0	39	31.6	48.4	0.346
		12	39	31.6	50	
CHOL [mmol/L]	Experienced *n* = 31	0	4.78	2.63	7.75	0.983
		12	4.65	3.04	6.14	
	Naïve *n* = 39	0	4.58	2.52	7.75	0.809
		12	4.58	2.73	7.02	

* statistically significant difference in Mann–Whitney U test.

**Table 3:** The frequency of fibrosis stages before (week 0) and after AbbVie’s 3D regimen (week 12).

METAVIR Score	Week 0 *n* (%)	Week 12 *n* (%)
F0–F1	33 (47.1)	42 (60)
F1	1 (1.4)	5 (7.1)
F1–F2	8 (11.4)	10 (14.3)
F2	4 (5.7)	0 (0)
F2–F3	1 (1.4)	0 (0)
F3	5 (7.1)	5 (7.1)
F3–F4	1 (1.4)	0 (0)
F4	15 (21.4)	8 (11.4)
Total	70 (100)	70 (100)

Abbreviations: F0–F4, stages of fibrosis in METAVIR scale, *n*: number of patients.

**Table 4:** Comparisons of non-invasive indirect biomarkers of liver fibrosis between treatment experience and treatment naïve group at point 0 and point 12.

Test	Treatment	Point 0	Comparison 1	Point 12	Comparison 1	Comparison 2	Cut-Off [Ref.]
APRI	Experienced	0.66 ± 0.53	Z = −0.827 *p* = 0.408	0.30 ± 0.26	Z = −1.382 *p* = 0.167	*p* = 0.003 *p* = 0.007	≤0.5 [15]
	Naïve	1.09 ± 2.71		0.26 ± 0.25			
GAPRI	Experienced	0.91 ± 0.83	Z = −0.943 *p* = 0.346	0.32 ± 0.36	Z = −1.305 *p* = 0.192	*p <* 0.001 *p <* 0.001	0.52 [16]
	Naïve	0.74 ± 0.77		0.19 ± 0.14			
FIB-4	Experienced	2.48 ± 2.04	Z = −1.459 *p* = 0.144	1.87 ± 1.63	Z = −2.093 *p* = 0.036 *	*p* = 0.421 *p* = 0.234	<1.45 [17]
	Naïve	2.80 ± 4.37		1.43 ± 1.28			
AP index	Experienced	5.32 ± 2.48	Z = −2.014 *p* = 0.044 *	5.17 ± 2.22	Z = −2.519 *p* = 0.012 *	*p* = 0.704 *p* = 0.816	<4 [18]
	Naïve	3.97 ± 2.81		3.59 ± 2.56			
HUI	Experienced	4.46 ± 2.37	Z = −1.648 *p* = 0.100	4.22 ± 1.97	Z = −1.795 *p* = 0.073	*p* = 0.733 *p* = 1.0	5.54 [19]
	Naïve	3.67 ± 2.19		3.55 ± 2.47			
FORNS	Experienced	6.08 ± 2.00	Z = −1.434 *p* = 0.152	5.32 ± 1.92	Z = −2.196 *p* = 0.028 *	*p* = 0.517 *p* = 0.060	<4.21 [20]
	Naïve	5.34 ± 2.62		4.19 ± 2.25			

Comparison 1 between experienced and naïve groups was performed with the Mann–Whitney U test between week 0 and week 12 and comparison 2 was performed with a chi-squared test for trends. * statistically significant difference. Cut-offs were taken from references given in square brackets.

**Table 5:** Correlation between FibroScan results and non-invasive indirect markers of liver fibrosis at point 0 and point 12 of the study.

Test	FibroScan at Point 0	FibroScan at Point 12
APRI	R = 0.744 * (*p* < 0.001)	R = 0.664 * (*p* < 0.001)
FIB-4	R = 0.736 * (*p* < 0.001)	R = 0.611 * (*p* < 0.001)
FORNS	R = 0.704 * (*p* < 0.001)	R = 0.647 * (*p* < 0.001)
AP index	R = 0.643 * (*p* < 0.001)	R = 0.590 * (*p* < 0.001)
HUI	R = 0.662 * (*p* < 0.001)	R = 0.561 * (*p* = 0.001)
GAPRI	R = 0.509 * (*p* < 0.001)	R = 0.701 * (*p* < 0.001)

R—Spearman correlation coefficient; * statistically significant correlation.

## Data Availability

Data sharing not applicable.

## Abbreviations

ALT: alanine aminotransferase; AST, aspartate aminotransferase; DAAs, direct-acting antiviral agents; DSV, dasabuvir; EMA, European Medicines Agency; GGT, γ-glutamyl transferase; LSM, liver stiffness measure; MCV, mean corpuscular volume; PEG INF + RBV, pegylated interferon-α and ribavirin; PLT, platelets; RT-PCR, reverse transcriptase polymerase chain reaction; SVR12, sustained virologic response after 12 weeks of therapy; VCTE, vibration-controlled transient elastography.
